# The Role of mTOR Inhibitors in COVID-19 Outcomes Among Heart Transplant Recipients

**DOI:** 10.3390/v18010029

**Published:** 2025-12-24

**Authors:** Agnieszka Kuczaj, Szymon Warwas, Mikołaj Tyrka, Błażej Skotnicki, Daniel Szymecki, Oliwia Jewuła, Szymon Pawlak, Piotr Przybyłowski, Tomasz Hrapkowicz

**Affiliations:** 1 Department of Cardiac, Vascular and Endovascular Surgery and Transplantology, Faculty of Medical Sciences in Zabrze, Medical University of Silesia in Katowice, Silesian Center for Heart Diseases, M.C. Skłodowskiej 9, 41-800 Zabrze, Poland; 2 Students’ Scientific Association, Faculty of Medical Sciences in Zabrze, Medical University of Silesia in Katowice, M.C. Skłodowskiej 9, 41-800 Zabrze, Poland

**Keywords:** heart transplantation, mTOR inhibitors, COVID-19

## Abstract

Background: Heart failure (HF) remains a major global health challenge, with orthotopic heart transplantation (OHT) serving as the gold-standard therapy for end-stage disease. Chronic immunosuppression required to prevent graft rejection increases the risk of infections and malignancies. The COVID-19 pandemic underscored the particular vulnerability of transplant recipients to severe SARS-CoV-2 infection. Specific immunosuppressive agents used in OHT patients may differentially affect SARS-CoV-2 infection. In particular, mTOR inhibitors may modulate viral replication and immune responses, potentially influencing disease severity. Objectives: This study evaluated the impact of immunosuppressive regimens—particularly mTOR inhibitors—on COVID-19 outcomes in heart transplant recipients, comparing mTOR-based therapy (with or without calcineurin inhibitors, CNIs) to non-mTOR-based regimens. Methods: This single-center retrospective observational study included 556 orthotopic heart transplant recipients (76.3% male; median age, 58 years) followed from March 2020 to March 2024. To compare patients receiving mTOR inhibitors with similar non-mTOR recipients, 3:1 propensity score matching was performed based on age, sex, and body mass index. Among the study population, 88 patients (15.8%) received mTOR inhibitors (everolimus or sirolimus), of whom 66 were concomitantly treated with calcineurin inhibitors and 22 without. Data were obtained from the National Health Fund database and clinical follow-ups. Results: Overall mortality was 13.5%, and COVID-19-related mortality 3.2%. COVID-19 incidence was 33% in the mTOR group versus 36.7% in the non-mTOR group (*p* = 0.52). Hospitalization rates were 3.4% and 6.4% (*p* = 0.29), respectively. All-cause mortality was higher among mTOR users (21.6% vs. 11.7%, *p* = 0.02), especially in the mTOR+CNI subgroup. Notably, no COVID-19-related deaths occurred in the mTOR CNI-free group. Conclusions: mTOR-based immunosuppression was non-inferior to standard therapy for COVID-19 outcomes. The absence of COVID-19-related deaths in patients on mTOR CNI-free regimens suggests potential protective effects that merit further investigation.

## 1. Introduction

Heart failure (HF) represents an increasingly significant global health concern, imposing a substantial economic burden on healthcare systems. In cases of end-stage heart failure, orthotopic heart transplantation (OHT) remains the gold standard of treatment, significantly improving quality of life and prolonging survival in appropriately selected candidates [1,2,3].

The principal challenge following transplantation is the risk of acute and chronic allograft rejection, driven by immune responses directed against donor antigens. To mitigate this risk, immunosuppressive therapy is implemented, typically consisting of calcineurin inhibitors (tacrolimus, cyclosporine), antiproliferative agents (mycophenolate mofetil, azathioprine), mTOR inhibitors (everolimus, sirolimus), and glucocorticosteroids. Individualizing the immunosuppressive regimen is essential, as inadequate immune suppression increases the risk of rejection, whereas excessive immunosuppression may lead to nephrotoxicity, myelosuppression, and post-transplant diabetes mellitus. Immunosuppressive therapy is also associated with an elevated risk of infections, affecting up to 35% of patients in the early postoperative period [4]. Reduced immunologic surveillance also increases the risk of malignancies, some of viral origin, such as Epstein–Barr virus associated post-transplant lymphoproliferative disorder and skin cancers [5].

The advent of the COVID-19 pandemic has further highlighted the clinical implications of sustained immunosuppression in heart transplant recipients. Their compromised immune surveillance renders them disproportionately vulnerable to novel pathogens such as SARS-CoV-2, a member of the coronavirus family [6,7]. The infection may present asymptomatically or with flu-like symptoms including fever, cough, malaise, and myalgia [8]. In severe cases, COVID-19 can progress to respiratory failure, necessitating hospitalization and mechanical ventilation, and may culminate in septic shock and multi-organ failure [7].

Existing literature indicates that immunosuppression, which is indispensable in patients following OHT, contributes to a more severe COVID-19 clinical course and increased complication rates [9,10]. Modulation of the inflammatory response achieved through immunosuppressive therapy, particularly with mTOR inhibitors, may alter the inflammatory reaction during viral infections such as SARS-CoV-2. In patients with post-inflammatory or autoimmune etiologies of heart failure being a cause of heart transplantation—which represent relatively common causes of dilated cardiomyopathy—the inflammatory response triggered by SARS-CoV-2 infection may differ from that observed in patients with other underlying causes. This is especially relevant given that the majority of myocarditis cases are viral in origin [11,12]. It should also be noted that coronary artery disease, being a leading cause of heart transplantation, is likewise considered an inflammatory condition [13]. Multimorbidity further exacerbates vulnerability, with coexisting conditions such as hypertension, diabetes mellitus, and chronic kidney disease associated with worse clinical outcomes [14].

Discovered in the 1970s and 1980s, calcineurin inhibitors (CNIs)—cyclosporine A and tacrolimus—revolutionized transplant immunosuppression [15]. These agents block interleukin-2 (IL-2) production, a cytokine critical for T-cell activation and proliferation [16]. Cyclosporine A forms a complex with cyclophilin to inhibit calcineurin, thereby preventing the nuclear translocation of NFAT and suppressing IL-2 gene transcription [17]. Tacrolimus acts similarly, forming a complex with FKBP12 to inhibit the same pathway [18].

mTOR inhibitors are utilized across various transplant and non-transplant contexts. In cardiac allograft vasculopathy, they are particularly advantageous due to their ability to inhibit vascular smooth muscle cell proliferation, reduce neutrophil adhesion, and suppress VEGF expression—mechanisms that contribute to vascular remodeling prevention [19,20,21]. These agents are also instrumental when CNIs must be reduced or avoided due to nephrotoxicity, offering nephroprotective benefits while maintaining immunosuppressive efficacy [21,22,23]. In cases of recurrent rejection, incorporating an mTOR inhibitor can enhance immunomodulation and therapeutic response [24].

The antiproliferative properties of mTOR inhibitors also confer antineoplastic activity, which is beneficial in reducing the incidence of post-transplant malignancies [23,25,26]. Mechanistically, these agents suppress mTOR kinase activity, leading to cell cycle arrest in the G1 phase and reduced proliferation of T and B lymphocytes [27,28]. Unlike CNIs, they do not directly inhibit cytokine gene transcription [27]. Additionally, mTOR pathway modulation appears to influence immune responses during viral infections. Notably, reduced CMV reactivation has been observed in patients receiving these agents [21,29].

Mycophenolate mofetil (MMF), a prodrug of mycophenolic acid, is commonly used as an adjunct to CNI- or mTOR-based regimens. It suppresses de novo purine synthesis in lymphocytes, thereby inhibiting both T- and B-cell proliferation and reducing cellular and humoral rejection. MMF usage is associated with decreased risk of graft vasculopathy and improved graft survival [30]. However, its administration also correlates with increased lymphopenia and heightened susceptibility to opportunistic infections [31].

The immunomodulatory effects of these agents and the diverse mechanisms by which they act may significantly influence the course of SARS-CoV-2 infection [32]. Reports suggest that immunosuppressive regimens including MMF are associated with higher mortality among transplant recipients diagnosed with COVID-19 compared to those using mTOR-based protocols [33]. As a result, several studies recommend dose reduction or temporary discontinuation of MMF during active infection [34,35].

In the context of the COVID-19 pandemic, the potential benefits of mTOR inhibition are currently under investigation. Emerging data indicate that mTOR blockade may limit viral replication through induction of autophagy, suppression of viral protein synthesis, and inhibition of host cell proliferation—mechanisms that may collectively reduce disease severity [36,37]. At the same time, concerns remain regarding their immunosuppressive properties, particularly the inhibition of lymphocyte proliferation, which could impair antiviral immunity [28]. Nevertheless, current evidence suggests that mTOR inhibitors influence the course of COVID-19 through multiple pathways, including regulation of inflammatory responses and cellular metabolism. These observations underscore the need for further research to clarify the therapeutic potential and limitations of these agents during viral pandemics.

This study aims to evaluate the role of immunosuppressive agents, with a particular focus on mTOR inhibitors, in shaping the clinical course of COVID-19 among heart transplant recipients. Special attention will be given to comparing outcomes between patients receiving mTOR inhibitors with CNIs and mTOR CNI-free regimens. Findings from this analysis may offer valuable insights for optimizing immunosuppressive strategies during the COVID-19 pandemic and in future viral outbreaks.

## 2. Methods

This single-center retrospective observational study was conducted to assess the role of mTOR inhibitor usage in heart transplant recipients during the COVID-19 pandemic. The observation period spanned from March 2020 to March 2024. A total of 556 heart transplant recipients who underwent OHT between 6 December 1992, and 25 June 2022, and whose follow-up included the COVID-19 pandemic period were included in the study and monitored at the transplant clinic. Patient information was gathered from the National Health Fund database, through hospital admissions, in-home medical visits, and via telephone consultations. Patient outcomes were compared between those treated with mTOR inhibitors and those managed on non-mTOR regimens. To reduce confounding due to baseline differences between groups, propensity score matching 3:1 was performed. Propensity scores were estimated based on age, sex, and body mass index. Covariate balance after matching was assessed using standardized mean differences. Detailed results of these analyses are provided in the Supplementary Materials. All subsequent analyses were conducted in the propensity score-matched cohort. Analyses of the unmatched cohort are reported in the Supplementary Materials. Within the mTOR cohort, results were further stratified into groups according to concomitant CNI therapy (mTOR+CNI, and mTOR CNI-free).

The study was conducted in accordance with internationally accepted ethical guidelines, including the Declaration of Helsinki. Ethical approval was obtained from the Bioethics Committee at the Medical University of Silesia (Decision No. PCN/CMN/0022/KB1/30/21).

In terms of COVID-19 diagnosis, patients were classified as infected if they met any of the following criteria: a positive result from a reverse transcription–polymerase chain reaction (RT-PCR) test, a positive SARS-CoV-2 antigen rapid test on a nasopharyngeal swab, or a history of clinical symptoms consistent with COVID-19 accompanied by a presence of anti-SARS-CoV-2 antibodies without prior immunization.

## 3. Statistical Analysis

The continuous variables were expressed as medians with interquartile ranges (IQR), due to the assessment of normality of distribution in the Shapiro–Wilk test. Categorical variables were presented as counts and percentages. The groups were compared with the implementation of Chi-square or Fisher’s exact tests for categorical variables, as appropriate, and the Mann–Whitney U test was used for continuous variables. The Cox proportional hazards model was used to estimate the risk of death due to COVID-19 and all-cause mortality in the subsequent study groups. Proportional hazards assumption was assessed using Schoenfeld residuals. Proportional hazards assumption was assessed using Schoenfeld residuals; the detailed results can be found in the Supplementary Materials. A two-sided *p*-value < 0.05 was considered statistically significant. All calculations were performed using STATISTICA software (version 13.3, TIBCO Software Inc., San Ramon, CA, USA).

## 4. Results

Among 556 heart transplant recipients, 424 (76.3%) were male. The median age was 58 years (IQR: 44–66). A total of 282 patients (50.7%) had diabetes mellitus, and 429 (77.2%) had arterial hypertension. Obesity was identified in 127 patients (22.8%). The median serum creatinine level was 114 µmol/L (IQR: 91–146).

Regarding heart failure etiology, the most common cause was dilated cardiomyopathy, observed in 268 patients (48.2%), followed by ischemic cardiomyopathy in 192 (34.5%). Other etiologies included hypertrophic cardiomyopathy in 25 patients (4.5%), restrictive cardiomyopathy in 18 (3.2%), arrhythmogenic right ventricular cardiomyopathy in 7 (1.3%), valvular cardiomyopathy in 8 (1.4%), peripartum cardiomyopathy in 6 (1.1%), and other or unspecified types in 32 (5.8%).

The median time from OHT to COVID-19 diagnosis was 1478 days (IQR: 509–4421). The median follow-up time post-OHT was 3284 days (IQR: 1777–5882).

Overall all-cause mortality was 13.5%, while COVID-19-related mortality accounted for 3.2%. Cardiac allograft vasculopathy was diagnosed in 115 patients (20.7%).

Regarding immunosuppressive therapy, tacrolimus was the primary CNI used in 477 recipients (85.8%), while cyclosporine was administered to 56 (10.1%). Monotherapy with tacrolimus was noted in 120 cases (21.6%) and cyclosporine in 17 cases (3.1%). Remaining immunosuppressants included mycophenolate in 350 patients (62.9%), everolimus in 70 (12.6%), and sirolimus in 18 (3.2%). Among patients receiving mTOR inhibitors, 19/88 (21.6%) also received mycophenolates. In the CNI-based regimen group, 63% of patients received mycophenolates. During COVID-19 infection, mycophenolates were tapered or discontinued at the discretion of the treating physician.

According to protocol, corticosteroids were continued for up to one year post-OHT in 90 patients (16.2%). Additionally, 95% of the cohort received statins, and 96% were treated with acetylsalicylic acid (ASA). Antiviral prophylaxis with valganciclovir was maintained until day 110 post-OHT, followed by six months of trimethoprim-sulfamethoxazole for antibacterial coverage.

In total, 88 patients (15.8%) received mTOR inhibitor therapy (everolimus or sirolimus). Of these, 66 (11.9%) were treated with a combination of mTOR inhibitors and CNIs (tacrolimus or cyclosporine), while 22 (4%) received an mTOR CNI-free regimen. Moreover, 87 cases (15.6%) of therapy conversion to a combination of CNI and mTOR inhibitors were observed. Thirteen of these (14.9%) resulted from diagnosed chronic kidney disease, 37 (42.5%) from cardiac allograft vasculopathy, 11 (12.6%) following acute cellular rejection, 4 (4.6%) due to cytomegalovirus infection, 7 (8.0%) caused by malignancies, and 15 (17.2%) were based on the physician’s decision for various other reasons.

Accordingly, we proceeded with a propensity-score-matched analysis to provide adjusted comparisons between the mTOR and non-mTOR groups. The mean age in the mTOR group was 60.5 years (IQR: 47–67.5) compared to 61 years (IQR: 48–67) in the non-mTOR group (*p* = 0.86). COVID-19 incidence was 33% (29/88) in the mTOR group versus 36.7% (97/264) in the non-mTOR group (*p* = 0.52). Hospitalization rates were 3.4% (3/88) for mTOR patients and 6.4% (17/264) in the non-mTOR group (*p* = 0.29). All-cause mortality during the observation period was higher in the mTOR group at 21.6%, compared to 11.7% in the non-mTOR group (*p* = 0.02). No statistically significant differences in sex distribution were observed between the groups, with males comprising 86.4% of patients in the mTOR group and 86.0% in the non-mTOR group (*p* = 0.93). Furthermore, the groups differed significantly in time from OHT to COVID-19 infection (3322 vs. 1451 days; *p* = 0.03) and in follow-up duration post-OHT (4912 vs. 3175 days; *p* < 0.001). Detailed comparisons between the mTOR and non-mTOR groups are presented in Table 1.

To assess all-cause and COVID-19-related mortality across various subgroups, Cox proportional hazards models were constructed. The combination of mTOR inhibitors and CNIs was associated with a more than twofold increase in the risk of all-cause mortality. Detailed results are presented in Table 2.

**Figure 1:**
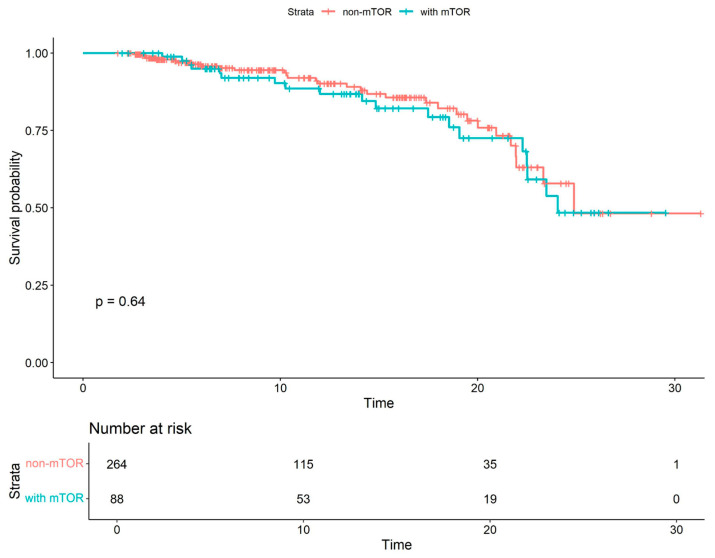
Kaplan–Meier survival curves for patients receiving mTOR inhibitors vs. non-mTOR-based therapy. The analysis included only patients who were under follow-up during the COVID-19 pandemic.

Kaplan–Meier survival curves were generated to estimate survival probabilities in patients receiving mTOR inhibitors and non-mTOR-based therapy. The difference in overall survival between these groups did not reach statistical significance (see Figure 1).

**Figure 2:**
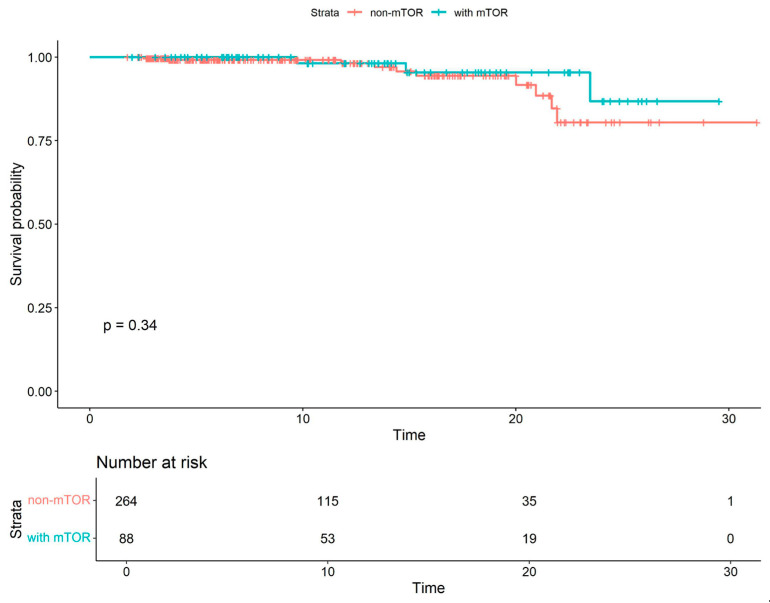
COVID-19-specific Kaplan–Meier survival curves for patients receiving mTOR inhibitors and non-mTOR-based therapy. The analysis included only patients who were under follow-up during the COVID-19 pandemic.

A separate Kaplan–Meier survival analysis was conducted to estimate COVID-19-specific survival in patients receiving mTOR inhibitors versus non-mTOR-based therapy. The difference in survival between the two groups was not statistically significant (see Figure 2).

**Figure 3:**
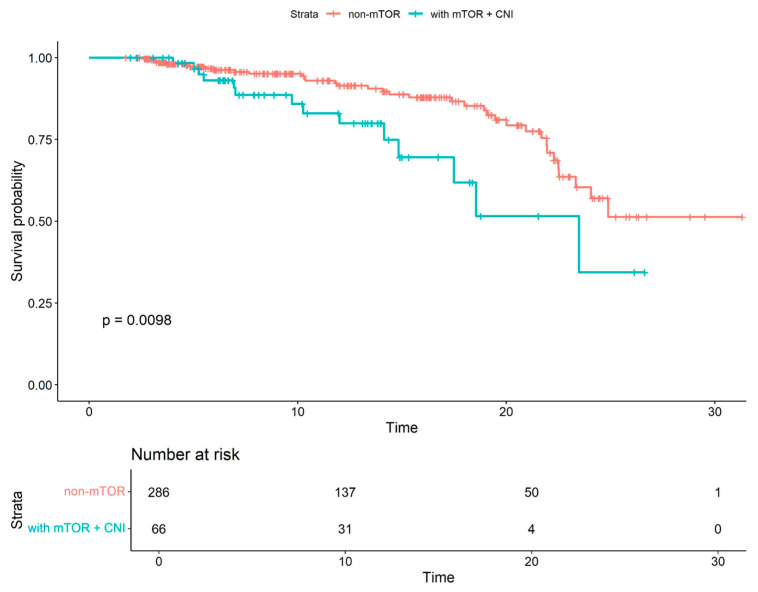
Kaplan–Meier survival curves comparing non-mTOR-based therapy and mTOR+CNI combination therapy. The analysis included only patients who were under follow-up during the COVID-19 pandemic.

**Figure 4:**
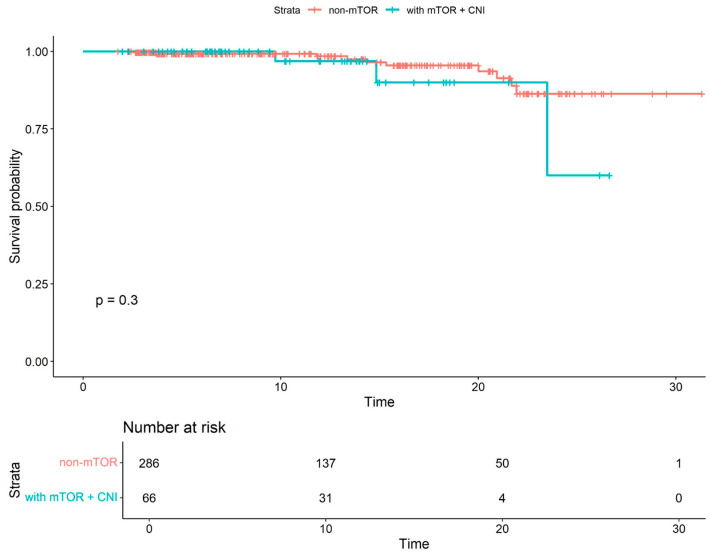
COVID-19-specific Kaplan–Meier survival curves for non-mTOR-based therapy and mTOR+CNI combination therapy. The analysis included only patients who were under follow-up during the COVID-19 pandemic.

Kaplan–Meier survival analyses were subsequently conducted for subgroups to compare survival probabilities between patients receiving non-mTOR-based therapy and those receiving mTOR combined with CNI (Figure 3), as well as between patients receiving non-mTOR-based therapy and those receiving the mTOR CNI-free regimen. In both comparisons, the differences in survival probabilities were statistically significant. Patients treated with mTOR inhibitors and CNIs exhibited lower survival probabilities (*p* = 0.01), whereas those receiving mTOR CNI-free regimen demonstrated improved survival outcomes (*p* = 0.02). Additionally, COVID-19-specific survival was analyzed between the non-mTOR-based therapy group and the mTOR+CNI combination therapy group (Figure 4), showing no significant survival differences between groups (*p* = 0.3). Statistical comparison between the non-mTOR-based therapy group and the mTOR CNI-free group was not feasible, as no COVID-19-related deaths were observed in the latter group.

## 5. Discussion

In the present study, an mTOR-based regimen in patients treated under pandemic circumstances was found to be non-inferior to the standard regimen consisting of CNIs and mycophenolates.

The use of mTOR inhibitors as a primary immunosuppressive regimen is limited by their adverse-effect profile. During the early post-transplant stage, the use of these agents is not advised, as they are associated with impaired wound healing and the development of exudates. At the same time, in monotherapy these agents are considered less potent than CNIs, and their introduction in the early post-transplant period may lead to graft rejection [38].

In the later post-transplant period, adverse effects like peripheral edema, skin rash, and oral ulcerations may lead to poor patient adherence to the therapy. Among the risks associated with the use of this drug, hypersensitivity pneumonitis related to mTOR inhibition is also reported [39,40]. As an antiproliferative agent mTOR inhibitors are advised in graft vasculopathy and cancers [21]. Although mTOR inhibitors may exacerbate pre-existing proteinuria, their use can also be justified in cases of chronic kidney disease progression secondary to CNI-induced nephrotoxicity. Replacing mycophenolates with mTOR inhibitors while concomitantly lowering the dose of CNIs, or converting a CNI-based regimen to an mTOR inhibitor-based regimen with or without mycophenolates, may help slow the progression of renal disease.

As previously mentioned, due to multiple concerns regarding mTOR inhibitors, patients received these drugs primarily due to complications in the post-transplant course occurring upon basal immunosuppression consisting of CNIs and mycophenolates, rather than as first-line immunosuppressive therapy. These complications—such as graft vasculopathy, rejection, and renal dysfunction—are generally considered to be associated with poorer patient prognosis and accumulate in some patients over time [38].

Analyzing our population, patients who were prescribed mTOR inhibitors had a longer time since transplantation than the rest of the study population. According to this fact, these patients should be more prone to adverse events. Nevertheless, under conditions of long-term, stable immunosuppressive regimens containing mTOR inhibitors, these patients as a whole did not demonstrate poorer outcomes with respect to COVID-19 survival.

Accumulated data on COVID-19 indicate that dysregulation of the immune response plays a key role in the pathophysiology of severe cases of the disease [41,42]. Accordingly, it appears that drugs and substances modulating the immune response, administered both for the treatment of COVID-19 and for other indications, such as chronic use for immunosuppression in organ transplant recipients, may significantly influence the course of COVID-19 [43].

mTOR signaling is necessary for SARS-CoV-2 replication. Inhibition of mTORC1 by mTOR inhibitors like sirolimus and everolimus promotes autophagy of infected hosts’ cells and inhibits translation of important viral proteins (proteases, structural proteins) [44].

Pharmacological inhibition of mTOR—in particular the mTORC1 blockade mediated by everolimus and sirolimus—may contribute to differential immunological effects in COVID- 19, characterized by a suppression of T-cell proliferation, potentially attenuating the cytokine storm, while simultaneously maintaining lymphocyte Treg expansion and function, thereby limiting hyperreactivity during the critical stage of the disease [45].

In vitro studies, likewise, have demonstrated that inhibition of the mTOR signaling pathway via the Akt inhibitor MK-2206 suppresses SARS-CoV-2 replication [46].

Omarjee et al. proposed that administering mTOR inhibitors at an early stage of the cytokine storm could modulate the secretion of proinflammatory interleukins, TNFα, metalloproteinases and growth factors, thereby potentially preventing progression to severe COVID-19 [47].

In a study reporting off-label sirolimus use, continuous administration of sirolimus before and during infection was associated with a less severe course of COVID-19 and no occurrence of long COVID, compared to the group without rapamycin administration, despite the fact that rapamycin users were older. The authors themselves, however, approach the results of their work with caution due to the varying duration and doses of sirolimus used [48].

The potentially beneficial effect of mTOR therapy on the course of infection may also be explained through mechanisms connected with immunization. A considerable proportion of the study population had received vaccination, which may have contributed to the observed outcomes. In support of this, Perkins et al. reported that kidney transplant recipients maintained on mTOR-based immunosuppression demonstrated enhanced memory cell formation following vaccination compared with those receiving standard-of-care immunosuppression [49].

An interesting observation is that in the analyzed period of the COVID-19 pandemic, the subgroup of patients not receiving calcineurin inhibitors exhibited better total survival than those undergoing immunosuppression based on calcineurin inhibitors. It could be speculated that these individuals remaining on non CNI based immunosuppression regimen were individuals with better graft tolerance and for whom a less intensive immunosuppressive regimen (without CNIs) was sufficient. However, the study was designed to compare patient survival during the pandemic rather than the benefits of introducing specific drug classes and such far-reaching conclusions are difficult to draw. A particularly interesting observation was that among patients receiving immunosuppression without calcineurin inhibitors, no deaths attributable to COVID-19 were recorded. The results of our study are consistent with research conducted in the kidney transplant population: in a study of 371 kidney recipients, Pinchera et al. demonstrated that the use of mTOR inhibitors was associated with a lower incidence of moderate or severe forms of SARS-CoV-2 infection [50]. This may be related to the fact that CNI-free immunosuppression is relatively weak and thus allows for a more effective host defense against viral infection. Alternatively, the potential antiviral properties of mTOR inhibitors might play a role. However, these results are promising, this requires further investigation in the context of COVID-19, as drawing conclusions from these small patient cohorts would be premature.

In our investigated group, the combination of mTOR inhibitors and CNI was particularly frequently used in patients with vasculopathy, cellular rejection, and renal dysfunction—factors that represent independent risk factors for mortality in heart transplant recipients. It is therefore unsurprising that these patients exhibited higher all-cause mortality compared with the remaining cohort. Interestingly, however, this significant difference in mortality does not extend to deaths attributable to COVID-19. While firm conclusions about the potential benefits of mTOR inhibitors in COVID-19 cannot yet be drawn, the observation is intriguing and needs further investigation.

## 6. Conclusions

No significant differences in COVID-19 outcomes were observed between mTOR and non-mTOR regimens. The lack of deaths in the mTOR-CNI-free group is notable but requires cautious interpretation due to the small sample size. The underlying mechanisms of this observation warrant further investigation.

## Figures and Tables

**Table 1:** Comparison between matched patients receiving immunotherapy without mTOR inhibitors versus patients with mTOR inhibitors.

	All Cohort (N = 352)	Patients Without mTOR Inhibitors (N = 264)	Patients with mTOR Inhibitors (N = 88)	*p* Value
COVID-19 incidence, n (%)	126 (35.8)	97 (36.7)	29 (33)	0.52
COVID-19 hospitalization, n (%)	20 (5.7)	17 (6.4)	3 (3.4)	0.29
COVID-19-related death, n (%)	13 (3.7)	10 (3.8)	3 (3.4)	0.87
COVID-19 vaccinated, n (%)	276 (78.4)	202 (76.5)	74 (84.1)	0.13
All-cause mortality, n (%)	50 (14.2)	31 (11.7)	19 (21.6)	0.02
Male sex, n (%)	303 (86.1)	227 (86)	76 (86.4)	0.93
Diabetes mellitus type 2, n (%)	192 (54.5)	146 (55.3)	46 (52.3)	0.62
Arterial hypertension, n (%)	283 (80.4)	214 (81.1)	69 (78.4)	0.59
Obesity, n (%)	89 (25.3)	68 (25.8)	21 (23.9)	0.72
Age (years)	61 (48–67)	61 (48–67)	60.5 (47–67.5)	0.86
Creatinine level (mg/dL)	116 (92.8–148)	117 (94–147)	116 (89.5–148)	0.54
COVID-19 antibodies level (BAU/mL)	30.9 (0.4–250)	22.2 (0.4–250)	70.48 (2.11–250)	0.06
Body mass index (kg/m^2^)	26.2 (24–30.1)	26.2 (24.1–30.1)	26.28 (23.9–29.9)	0.92
Cardiac allograft vasculopathy, n (%)	70 (19.9)	56 (21.2)	14 (15.9)	0.28
Follow-up time since OHT (days)	3457 (1903–6005)	3175 (1658–5866)	4912 (2436.5–6913)	<0.001

**Table 2:** Cox proportional hazards models for all-cause and COVID-19-related mortality among subgroups receiving different regimens of mTOR therapy.

	Hazard Ratio (HR)	95% Confidence Interval (CI)	*p*-Value
Combined mTOR all-cause mortality	1.15	0.64–2.05	0.64
Combined mTOR COVID-19-related mortality	0.54	0.15–1.98	0.35
mTOR+CNI all-cause mortality	2.24	1.2–4.2	0.01
mTOR+CNI COVID-19-related mortality	1.97	0.53–7.3	0.31
mTOR CNI-free all-cause mortality *	0.43	0.17–1.1	0.08

* Proportional hazards assumption using Schoenfeld residuals not met.

## Data Availability

The data presented in this study are available on request from the corresponding author.

## Supplementary Materials

The following supporting information can be downloaded at: https://www.mdpi.com/article/10.3390/v18010029/s1, Figure S1: Study flowchart; Table S1. Comparison between the whole cohort of patients receiving immunotherapy without mTOR inhibitors versus patients with mTOR inhibitors; Table S2: Initial Cox proportional hazards models for all-cause and COVID-19-related mortality among subgroups receiving different regimens of mTOR therapy; Table S3: Results of proportional hazards assumption testing using Schoenfeld residuals. Table S4: Results of propensity score matching.

## Abbreviations

ASA	Acetylsalicylic acid
CNI	Calcineurin inhibitor
CMV	Cytomegalovirus
EBV	Epstein–Barr virus
HF	Heart failure
HFA	Heart Failure Association
IL-2	Interleukin-2
MMF	Mycophenolate mofetil
OHT	Orthotopic heart transplantation
PTLD	Post-transplant lymphoproliferative disorder
