# Supplementary material for: The Role of mTOR Inhibitors in COVID-19 Outcomes Among Heart Transplant Recipients
Source: Viruses. 2025 Dec 24;18(1):29. doi: 10.3390/v18010029 (PMC12846580; doi:10.3390/v18010029)
Supplement: Supplementary file 1 [file viruses-18-00029-s001.zip › viruses-4023563-supplementary.pdf]

Supplementary Materials:

Figure S1. Study flowchart

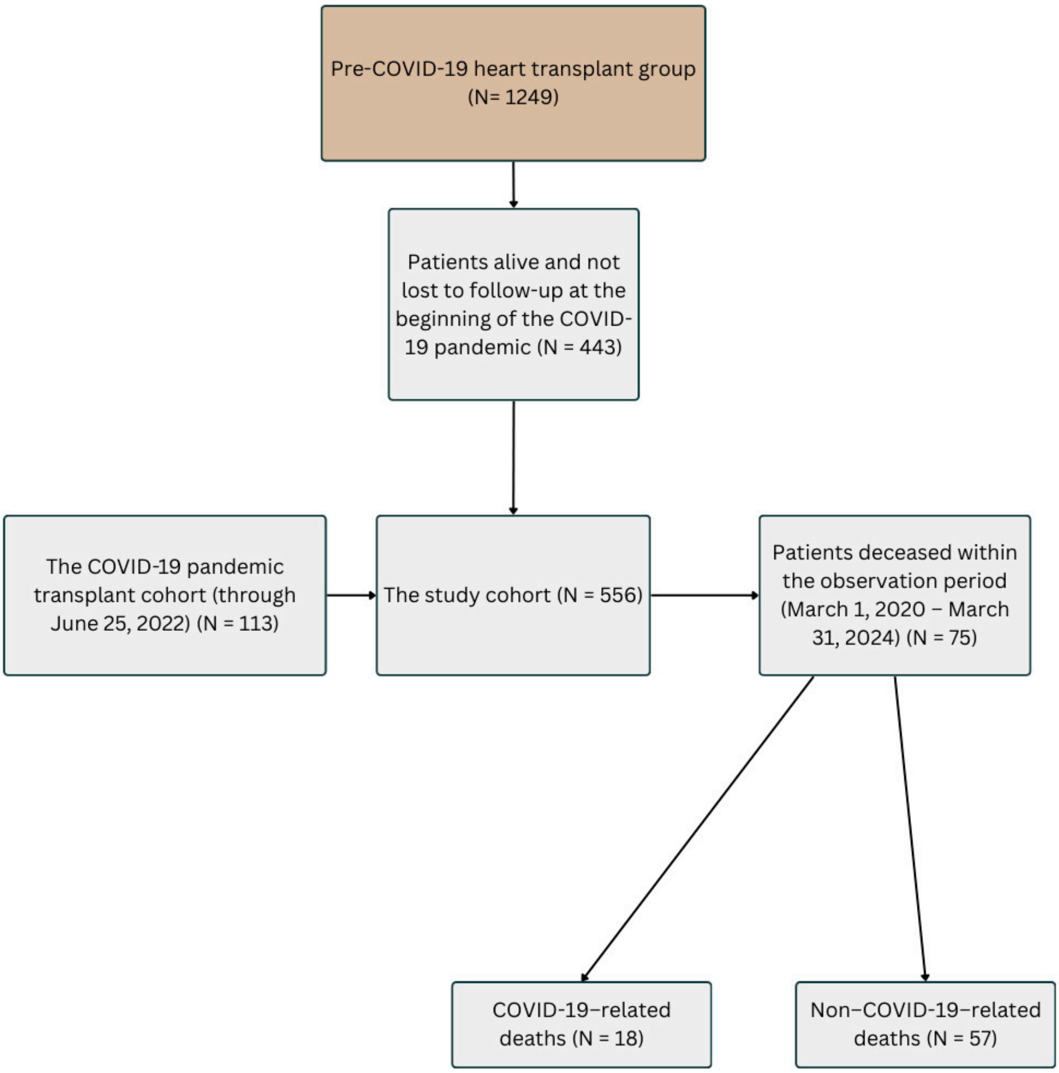

Table S1. Comparison between the whole cohort of patients receiving immunotherapy without mTOR inhibitors versus patients with mTOR inhibitors.

	All cohort (N=556)	Patients without mTOR inhibitors (N=468)	Patients with mTOR inhibitors (N=88)	P value
COVID-19 incidence, n (%)	189 (34)	160 (34.2)	29 (33)	0.82
COVID-19 hospitalization, n (%)	33 (5.9)	30 (6.4)	3 (3.4)	0.40
COVID-19-related death, n (%)	18 (3.2)	15 (3.2)	3 (3.4)	0.82
COVID-19 vaccinated, n (%)	432 (77.7)	358 (76.5)	74 (84.1)	0.13
All-cause mortality, n (%)	75 (13.5)	56 (12)	19 (21.6)	0.02
Male sex, n (%)	424 (76.3)	348 (74.4)	76 (86.4)	0.02
Diabetes mellitus type 2, n (%)	282 (50.7)	236 (50.4)	46 (52.3)	0.76
Arterial hypertension, n (%)	429 (77.2)	360 (76.9)	69 (78.4)	0.84
Obesity, n (%)	126 (22.7)	105 (22.4)	21 (23.9)	0.77
Age (years)	58 (44-66)	57.5 (43-65.5)	60.5 (47-67.5)	0.07
Creatinine level (mg/dl)	114 (91-146)	113 (91-146)	116 (89.5-148)	0.93
COVID-19 antibodies level (BAU/ml)	49.82 (0.4-250)	45.62 (0.4-250)	70.48 (2.11-250)	0.41
Body mass index (kg/m²)	26.15 (23.9-29.7)	26.12 (23.9-29.6)	26.28 (23.9-29.9)	0.74
Cardiac allograft vasculopathy, n (%)	115 (20.7)	101 (21.6)	14 (15.9)	0.23
Follow-up time since OHT (days)	3284 (1777-5882)	3098 (1620-5778.5)	4912 (2436.5-6913)	<0.001

Table S2. Initial Cox proportional hazards models for all-cause and COVID-19-related mortality among subgroups receiving different regimens of mTOR therapy.

	Hazard ratio (HR)	95% Confidence Interval (CI)	*P* - value

Combined mTOR all-cause mortality	1.19	0.706-2.013	0.51
Combined mTOR COVID-19-related mortality	0.69	0.197-2.386	0.55
mTOR+CNI all-cause mortality	2.2	1.218-3.959	0.009
mTOR+CNI COVID-19-related mortality	1.98	0.568-6.926	0.28
mTOR CNI-free all-cause mortality	0.48	0.192-1.211	0.12

Table S3. Results of proportional hazards assumption testing using Schoenfeld residuals.

	chisq	df	p
Combined mTOR all-cause mortality	0,0345	1	0,85
GLOBAL	0,0345	1	0,85
	chisq	df	p
Combined mTOR COVID-19-related mortality	0,0118	1	0,91
GLOBAL	0,0118	1	0,91
	chisq	df	p
mTOR+CNI all-cause mortality	0,254	1	0,61
GLOBAL	0,254	1	0,61
	chisq	df	p
mTOR+CNI COVID-19-re-lated mortality	1	1	0,32
GLOBAL	1	1	0,32
	chisq	df	p
mTOR CNI-free all-cause mortality	4,23	1	0,04
GLOBAL	4,23	1	0,04

Table S4. Results of propensity score matching.

Summary of Balance for All Data:										
	Means	Treated	Means	Control	Std Mean Diff,	Var, Ratio	eCDF Mean	eCDF Max		
distance		0,1712		0,1562	0,387	0,7218	0,097	0,1824		
age		57,0341		53,7687	0,2498	0,7238	0,0558	0,1248		
male sex		0,8636		0,743	0,3514	,	0,1206	0,1206		
BMI		26,8205		26,7226	0,0227	0,84	0,0172	0,0628		
Summary of Balance for Matched Data:										
	Means	Treated	Means	Control	Std Mean Diff,	Var, Ratio	eCDF Mean	eCDF Max	Std,	Pair Dist,
distance		0,1712		0,1708	0,0121	1,0276	0,0069	0,053		0,0198
age		57,0341		57,178	-0,011	1,0663	0,0191	0,053		0,4399
male sex		0,8636		0,8598	0,011	,	0,0038	0,0038		0,0552
BMI		26,8205		26,9734	-0,0356	0,8567	0,0168	0,0492		1,1263
Sample Sizes:										
	Control	Treated								
All	468	88								
Matched	264	88								
Unmatched	204	0								

Discarded	0	0								
